# Acute lung injury following exposure to nitric acid

**DOI:** 10.4103/0970-2113.56354

**Published:** 2009

**Authors:** T. K. Jayalakshmi, Samir Shah, Ivona Lobo, Abhay Uppe, Ankur Mehta

**Affiliations:** *Department of Chest Disease and Tuberculosis, Pad. Dr. D. Y. Patil Medical College and Hospital, Mumbai, India*

**Keywords:** Acute respiratory distress syndrome, nitric acid inhalation, steroids

## Abstract

We present a series of three cases of survival following inhalation of nitric acid fumes, which resulted in acute respiratory distress. Inhalation of nitric acid fumes and its decomposition gases such as nitrogen dioxide results in delayed onset of acute respiratory distress syndrome. Intensive respiratory management, ventilatory support, and steroids can help in survival.

## INTRODUCTION

Toxic effects on the lungs can be caused by inhalation of a wide range of gases and fumes. Inhalation injuries occur as a result of asphyxiation, local irritation, toxic absorption, or allergy.[1] Irritant gases when inhaled in high concentrations injure the respiratory tract by causing acute inflammation, the main site of injury depending on the solubility of the gases. More soluble gases exert maximal effect, more proximally in the respiratory tract; and less soluble gases like nitric acid fumes exert maximal effect more distally.[2]

Nitric acid fumes and its decomposition gases such as oxides of nitrogen can cause devastating illness. We are presenting a series of three cases of survival following exposure to nitric acid fumes.

## CASE REPORTS

### Case 1

A 30-year-old man, a nonsmoker, presented to the hospital casualty ward with complaints of acute-onset breathlessness since early morning. He had been cleaning a nitric acid container the previous evening in the chemical and fertilizer unit where he was employed.

**Figure 1:**
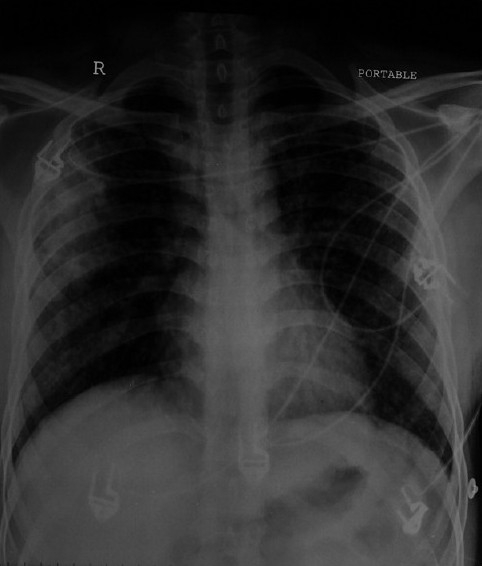
X ray chest PA view showing right upper lobe consolidation with bilateral mid and lower zone infiltrates

He was working with two co-workers when the incident occurred, and none of them was wearing any protective devices. They were exposed to fumes of nitric acid during cleaning. The duration of exposure was around 10 minutes, following which, immediately, he had some dry cough and a feeling of suffocation for a very short period. There was no eye, nasal, or skin irritation at that time. After around six hours, he developed progressive dyspnea, and he reported to the casualty ward in the morning with severe dyspnea. On arrival at the casualty ward, he had an SpO_2_ of 88%; pulse-112/min; B.P.-110/70 mmHg; RR-44/min; X-ray chest- bilateral upper, mid, and lower acinar opacities with right upper lobe consolidation [Figure 1].

He was admitted to the ICU and was given high-flow oxygen with nasal mask and rebreathing bag. However, he continued to worsen, with RR- 70/min and SpO_2_-91%. His arterial blood gases showed significant hypoxia, with pH-7.45, pO_2_-60 mmHg, and pCO_2_-28 mmHg. His complete blood counts, renal and hepatic parameters were normal.

**Figure 2:**
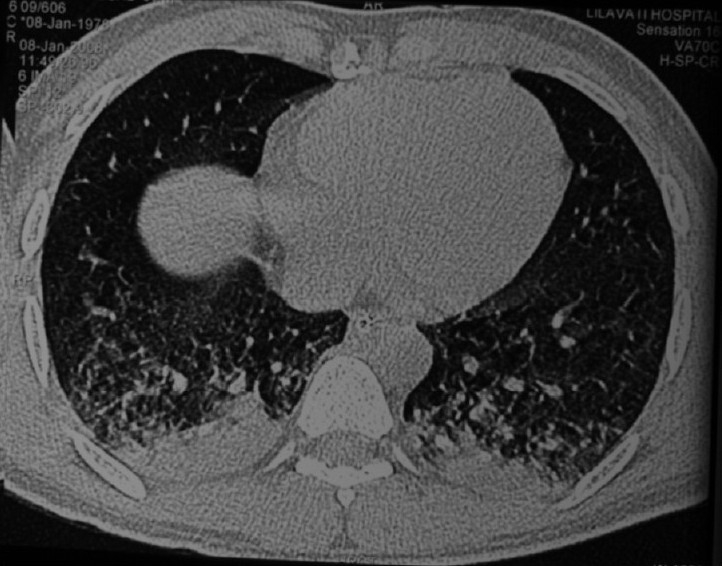
CT scan revealing B/L interstitial opacities with air trapping and with right upper lobe consolidation

His CT scan revealed bilateral interstitial opacities with air trapping and with right upper lobe consolidation [Figure 2].

A decision was made to intubate and initiate ventilatory support.

He was treated with methylprednisolone 125 mg and antibiotics and nebulized with bronchodilators and N-acetylcysteine (NAC).

The patient showed good clinical and radiological improvement. He was weaned off from the ventilator on day 4 and discharged after a week. Findings from his X-ray chest and pulmonary function test after one month were normal.

### Case 2

**Figure 3:**
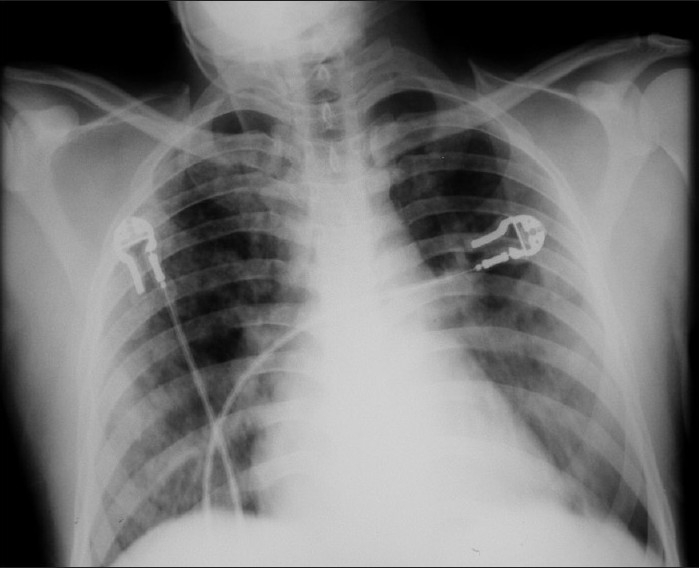
X-rays showing B/L upper and middle zone acinar opacities

A 35-year-old man with similar history of exposure as described above for case 1 presented with complaints of breathlessness and dry cough. His X-ray showed bilateral upper and middle zone acinar opacities [Figure 3]. He was tachypnoeic with RR-40/min, pulse-106/min, B.P.-90/60 mmHg but was maintaining saturation of around 95% with high-flow oxygen with rebreathing mask.

He was treated with high-flow O_2_, methylprednisolone, antibiotics, and nebulization with bronchodilators and NAC. He responded well.

His X-rays after two days showed good resolution of the interstitial opacities, and he was subsequently discharged on the fourth day.

### Case 3

A 28-year-old man, a nonsmoker, with a similar history of exposure as described for case 1 above presented with mild breathlessness and cough. He had mild hypoxia with tachypnoea 28/min, which responded to high-flow nasal O_2_ with mask, methylprednisolone, antibiotics, and nebulization with bronchodilators and N-acetylcysteine. He had normal X-ray chest on presentation.

His symptoms resolved within two days, after which he was discharged.

## DISCUSSION

Severe pulmonary sequelae due to inhalation of vapors and gases originating from nitric acid solutions have been divided into three categories: (1) immediate fatalities from very high concentrations, (2) delayed effects occurring within 48 hours, and (3) mild immediate effects followed by a short recovery period, but culminating in pneumonia (NIOSH, 1976; Hamilton and Hardy, 1974). Our patients had a delayed presentation, and the first patient probably belonged to the third category. Inhalation of gases and vapors originating from nitric acid can be extremely dangerous because they do not invoke a violent protective cough reflex such as occurs with chlorine and ammonia.[3,4]

Toxic exposure to nitrogen dioxide has generally occurred in four circumstances: Exposure to silo gases, welding, combustion of nitrogen-containing materials, and spills of nitric acid.[1]

In our case, the exposure resulted from addition of water for cleaning the nitric acid container, which was presumed to be empty. As a significant amount of nitric acid was still present in the container, it reacted with water, releasing nitric oxide and nitrous oxide fumes, which were inhaled by the cleaners. The acid vapor is usually colorless, whereas decomposition gases containing nitrogen dioxide are brown in color. Nitrogen dioxide irritant and relatively insoluble in water; therefore, it tends to bypass upper airway without any warning irritation of the eyes or nasopharynx.[5] In the moist mucoid environment of the lower respiratory tract, nitrogen dioxide dissolves and penetrates the bronchiolar and alveolar membranes, generating free radicals and nitric acid and nitrous acid, and causes acute lung injury.[6]

Following inhalation, the seriousness of effects depends more on the highest concentration reached, and not on the duration of inhalation. The first patient presented with life-threatening pulmonary edema almost 8 hours after the inhalation. This delayed effect has been well described in almost all cases of nitric acid and nitrogen dioxide inhalation injury reports.[1] Hajela *et al.,* have reported three deaths owing to rapidly progressing pulmonary edema of delayed onset after inhalation of fumes due to accidental nitric acid exposure.

The electron microscope examination in case study by Hajela *et al.,* suggested that inhaled nitrogen dioxide causes direct microvascular injury, leading to increased permeability. Neutrophils and serum-derived mediators have been implicated in developing pulmonary edema owing to inhalation. This probably explains why steroids appear to help.[7] Studies have shown that NAC is useful in acute lung injury and acute respiratory distress syndrome (ARDS) due to toxic gas exposure, hence this was used in our patients. Treatment by NAC has been shown to increase extracellular total antioxidant power and the total number of thiol molecules and also improve intracellular glutathione levels and the outcome for the patients. In conclusion, patients with ARDS are in a deficient oxidant-antioxidant balance, which can improve significantly if supplemented with NAC.[8]

Use of high-frequency ventilation and reduction of the mean and peak airway pressures have been recommended.[1,5]

The spectrum of illnesses in nitric acid inhalation injury is variable, and response severity is unpredictable. Survivors of high-level nitrogen dioxide exposure may experience a biphasic clinical response characterized by acute bronchospasm and laryngospasm; and then, development of pulmonary edema 8-24 hours later. after exposure the patient can develop bronchiolitis obliterans.[1] Most such cases resolve. As most of these incidents have been sporadic and accidental, follow-up of the cases is not thorough. All the three patients in our series followed up after 1 month. They were asymptomatic and had a normal chest X-ray and normal pulmonary function test (PFT). Further follow-up is not available as patients were migrant nonpermanent workers and they went back to their native place after the respective episodes.

## CONCLUSION

This case series highlights the serious health risk of nitric acid fumes exposure in an occupational setting. There is a crying need for increased awareness about the toxicity of nitric acid fumes among employees and employers at chemical units; as well as about the necessity of using protective devices, resulting in implementation of the same to prevent such exposures in future. Our patients had not had the benefit of use of protective mask or other devices and were fortunate to have survived the exposure.
